# Association of behavioral risk factors with self-reported and symptom or measured chronic diseases among adult population (18–69 years) in India: evidence from SAGE study

**DOI:** 10.1186/s12889-019-6953-4

**Published:** 2019-05-14

**Authors:** Sunita Patel, Faujdar Ram, Surendra Kumar Patel, Kaushalendra Kumar

**Affiliations:** 1 International Institute for Population Sciences (IIPS), Sunita Patel, Library Building, IIPS, Deonar, Govandi East, Mumbai, Maharashtra 400088 India; 2 Population Council, New Delhi, India; 3 Department of Public Health and Mortality Studies, International Institute for Population Sciences (IIPS), Mumbai, Maharashtra India

**Keywords:** Behavioral risk factors, Physical inactivity, Self-reported chronic diseases, Symptom or measured chronic diseases

## Abstract

**Background:**

The objective is to analyze the behavioral risk factors among the adult population and to identify the determinants of and their association with self-reported and symptom or measured chronic diseases in India.

**Methods:**

The study utilized data from the Study on Global Aging and Adult Health (SAGE), Wave 1 (2007). Logistic regression was applied to examine the association of self-reported and symptom or measured chronic diseases with behavioral risk factors and socioeconomic-demographic covariates.

**Results:**

The results show that the prevalence of the symptom or measured chronic diseases was higher (41.9%) than that of the self-reported chronic diseases (24.1%). The moderate and vigorous physical activity was less likely to be associated with self-reported depression, arthritis, and stroke, but more likely to be associated with the symptom or measured based arthritis and asthma compared to physical inactivity. Adequate intake of fruits and vegetables was significantly less likely to be associated with angina, COPD, and asthma; however, it was more than three times more likely to be associated (OR: 3.45; 95% CI: 1.99–5.97) with self-reported depression. Infrequent moderate alcohol drinking was statistically two times more associated (OR: 1.83; 95% CI: 1.04–3.21) with the symptom or measured based COPD than non-drinking. Likewise, any type of tobacco use was found to be about four times more associated (OR: 3.59; 95% CI: 1.07–12.13) with self-reported stroke. Both self-reported and symptom or measured hypertension, arthritis, and diabetes were associated with overweight, while hypertension was associated with obesity. Females and increased age came out as significant predictors of both self-reported and symptom or measured chronic diseases.

**Conclusion:**

The prevalence of chronic diseases and their association with BRFs and socioeconomic and demographic covariates differ markedly when assessed against self-reported criteria versus symptom or measured criteria. Adequate intake of fruits and vegetables is a crucial behavior that controls and delays the onset of chronic diseases. The study suggests that the National Program should remain focused on behavioral risk factors for maximum returns on health outcomes and that proper awareness and knowledge must be spread about healthy lifestyle behaviors throughout the country.

**Electronic supplementary material:**

The online version of this article (10.1186/s12889-019-6953-4) contains supplementary material, which is available to authorized users.

## Background

Chronic diseases, such as heart diseases, diabetes, chronic obstructive pulmonary diseases (COPD), stroke, depression, and hypertension, are the leading cause of morbidity and premature deaths. Worldwide, out of the total causes of death, chronic diseases caused 33.7% deaths in 1990, with the number of deaths increased to about 40.3% in 2016 [1]. The Institute for Health Metrics and Evaluation estimated the contribution of chronic diseases to Disability-Adjusted Life Years (DALY) at 19.8% in 2016. The global share of chronic diseases in terms of DALY has increased by 5.4% from 1990 to 2016. In India, deaths from chronic diseases increased from 23.2 to 39.9% during this same period [1], with the DALY share of chronic diseases standing at 21.1% for all ages in 2016, which was higher than the global DALY share [1]. Behavioral risk factors such as physical inactivity, poor nutrition, alcohol consumption, and tobacco smoking are a significant cause of chronic diseases. According to the World Health Organization (WHO), tobacco smoke causes 7.2 million deaths; alcohol causes 3.3 million; salt/sodium intake 4.1 million, and physical inactivity causes 1.6 million preventable deaths worldwide every year [2]. These behavioral risk factors account for 20–30% of all causes of mortality [2].

Non-communicable diseases (NCDs) caused 82% of premature (before 70 years age) deaths, which occurred in low and middle-income countries (LMICs) [3]. Among NCDs, cardiovascular diseases caused the highest number of deaths (37%), followed by cancers (27%), chronic respiratory diseases (8%), diabetes (4%), and others NCDs (24%) among those under the age of 70 years [3]. Many epidemiological studies have shown that chronic diseases emerge in the middle age after prolonged exposure to unhealthy lifestyle behaviors, including physical inactivity, unhealthy diet, and alcohol and tobacco consumption, all of which are considered major risk factors of chronic diseases [4–6]. The WHO (2014) estimates for India indicated that 12.1% were physically inactive, 4.3% consumed alcohol (per capita/liters), 12.9% were current tobacco smokers, 18.9% were overweight, and 3.8% were obese [3]. Chronic diseases share common behavioral and metabolic risk factors [7–9]. Physical inactivity increases 20–30% risk of all causes of mortality [10]. By contrast, regular physical activity reduces the risk of ischemic heart disease, diabetes, stroke, and cancer. Physical activity is a key determinant of the prevention of overweight and obesity [11]. Physically inactive populations report a higher mean number of chronic diseases, especially those associated with persistent symptoms [12]. A low intake of fish, fruits, and vegetables contribute to the risk of non-communicable diseases [13]. Alcohol consumption significantly increases the risk of respiratory infection and hypertension [6]. Alcohol consumption and physical inactivity have been found to be associated with overweight, abdominal obesity, hypertension, and hypertriglyceridemia [5]. Some studies have found that alcohol consumption has both beneficial and detrimental effects on diabetes, ischemic stroke, asthma, COPD, and heart diseases, depending on the pattern and volume of drinking [14–17]. On the other hand, usual drinking has significant cardioprotective effects in both males and females [18]. However, other studies indicate that alcohol consumption is associated with non-communicable diseases [19, 20]. A study by Bardach et al. (2017) found that alcohol drinking prevented CHD deaths but that it caused deaths from stroke [20]. Body Mass Index (BMI) and waist circumference have been found to have a significant relationship with blood pressure, total cholesterol level, and high-density lipoprotein (HDL) cholesterol, all of which are risk factors for chronic heart diseases [21]. Obesity has a positive association with asthma [22]. Obese people having asthma experience improved lung function and better health on losing weight [23]. Alcohol consumption accelerates the weight gain process among the moderate (non-daily) consumers and is associated with chronic diseases [24].

A study highlighted that the prevalence of self-reported chronic diseases underestimates the actual prevalence, which varies with social gradients. The prevalence is also influenced by information collection, sample size, recall bias, and quality of data collection, which are inherent limitations of a population survey [25]. Inadequate healthcare facility utilization and undiagnosed diseases are essential determinants of self-reported illnesses, which show an unequal distribution across the socioeconomic gradients. Therefore, there is a need to focus on a symptom or measured understanding of chronic diseases. Measuring chronic diseases is a big challenge in developing countries, where information systems remain poorly developed. Therefore, the use of symptom based or measured disease information may be more suitable because the lack of diagnosis or the lack of knowledge on the part of the patient minimizes the reporting bias [25, 26].

The literature reviewed and listed above suggests that behavioral risk factors (BRFs) are significantly associated with chronic diseases. However, the association of BRFs with the prevalence of self-reported and symptom or measured chronic disease has been poorly studied. There have been a few studies in the past on BRFs and specific chronic diseases, but they have been based on micro-level data from various pockets of India [5, 6, 12] and, as such, are not representative of the whole country. The present study is a step forward to explore the association of BRFs with self-reported and symptom or measured chronic diseases by socioeconomic-demographic gradients. The study on Global Ageing and Adult Health Survey (SAGE) Wave-1 data for India (2007) provides information on both self-reported and symptom based or measured chronic diseases and behavioral risk factors of chronic diseases [27]. This is a nationally representative survey conducted by the International Institute for Population Sciences (IIPS), Mumbai, in collaboration with the WHO, Geneva. The current study aims to analyze the distribution of BRFs among the adult population and to identify their association with the self-reported and symptom or measured chronic diseases in India.

## Methods

The present study utilized secondary data from the Study on Global Ageing and Adult Health (SAGE), Wave 1 of India collected in 2007. The survey interviewed respondents aged 18 years and above from six nationally representative states of India, namely Assam, Karnataka, Maharashtra, Rajasthan, Uttar Pradesh, and West Bengal. A total of 12,198 participants were selected for the interview, where because of refusal, uncertainty or residents moved elsewhere, the survey includes only 11,230 participants were considered for the present analysis. For our study, we considered only participants aged 18–69 years (*n* = 9839), of whom 4670 were in the 18–49 age group and 5169 were in the 50–69 age group. The study included those aged 18–69 years for the analysis because of the high morbidity and mortality among those aged less than 70 years [3]. In addition, older populations (aged 70 and above years) are less physically active due to the age factor and are more likely to experience several chronic conditions all at the same time [11].

The SAGE survey made use of multistage cluster sampling. Primary Sampling Units (PSUs) were drawn through the Probability Proportional to Size (PPS) sampling design. For the rural areas, a two-stage sampling procedure was followed, whereby in the first stage, villages were taken as the PSUs, and, in the next stage, 25 households from each village were selected systematically for the interviews. For the urban areas, a three-stage sampling design was used, whereby city wards were selected as the PSUs first. Next, from each city ward, two Census Enumeration Blocks were selected. Finally, 33 households were selected from each CEB for the survey. Overall, 285 villages and 93 CEBs were selected from each of the six states of India. The details of the sampling and other procedures are available in the SAGE report [27]. Face-to-face interviews were done to collect information on household and member characteristics such as education, health, health behaviors, chronic conditions, health care utilization, etc. The questions on the BRFs in the SAGE Survey followed the WHO STEPS guidelines for NCD surveillance. The SAGE Wave 2 data is not available in the public domain, so we used Wave 1 data, which provides information on major self-reported and symptom based or measured chronic diseases. The SAGE data is available in the public domain at the following link: http://apps.who.int/healthinfo/systems/surveydata/index.php/catalog/sage/about.

### Ethics approval and consent to participate

Our study analysis based on secondary dataset of Study on Global Ageing and Adult Health Survey (SAGE) Wave 1 (2007) in India. The SAGE data is available in public domain for research use and hence there is no ethical approval required for present research. The all ethical clearance and informed consent followed in SAGE Wave-1 survey and no personal identifiable information is available. Therefore, there was no requirement for ethical clearance or informed consent for this particular research work.

### Outcome variables

Chronic diseases: The SAGE survey provides information on eight major chronic diseases, namely stroke, angina, diabetes mellitus, chronic lung diseases (COPD), asthma, depression, hypertension, and arthritis. For this research study, we focused on all of these chronic diseases. We followed the WHO guidelines for the analysis [28]. The detailed description of the inclusion methods of the diseases is presented in Additional file 1: Table A1.

The SAGE survey asked each respondent two sets of questions on chronic conditions. The first set of questions was for self-reported chronic conditions and the second set was based on specific symptom based or measured chronic conditions.

Self-reported chronic diseases: The first set of questions asked was: “Have you ever been diagnosed with or told by a health professional that you have had a stroke, angina, diabetes mellitus, etc.?” The question was asked for each of the eight chronic diseases separately. The responses were coded as “0” for “no” and “1” for “yes” in the analysis.

Symptom or measured chronic diseases: The second set of questions was based on specific symptoms or measured health conditions. The WHO Rose Angina questionnaire was used for symptom-based assessment of angina pectoris in the SAGE study [26, 29]. Depression too was measured on the basis of a symptom-based assessment [26]. The assessment used was the World Mental Health Survey version of the Composite International Diagnostic Interview schedule [30], as part of which 18 questions on depression were posed to the respondents. COPD was identified on the basis of the performance of lung function as measured by a spirometer [29]. It was calculated with consideration for the age and the actual height of the respondents. The health function was categorized into four categories coded as “0” for “mild”, “1” for “moderate”, “2” for “severe”, and “3” for “very severe”. The very severe category coded as “1” for having COPD and “0” for “having no COPD” was included in the analysis [31]. Hypertension was determined based on three sitting blood pressure readings taken at one minute’s interval. The individuals were categorized as hypertensive if the average systolic blood pressure (SBP) exceeded 140 mmHg and the diastolic blood pressure (DBP) exceeded 90 mmHg [26]. Asthma, arthritis, and stroke were identified based on their respective symptoms. For diabetes, respondents were asked if they had high blood sugar levels and had been taking insulin for regulating their blood sugar levels. On the basis of all this information, symptom-based or measured chronic diseases were identified (Additional file 1: Table A.1).

### Exposure variables

Behavioral risk factors including physical activity, nutrition, alcohol consumption, and tobacco use were taken as the key exposure variables in this study.

Behavioral Risk Factors (BRFs):

Physical activity: For physical activity, the questions assessed the duration of activity (minutes and/or hours) on a typical day. The duration of activity included: i) activities at the workplace (work such as paid or unpaid work, household chores, harvesting food/crops, fishing or hunting for food, providing care, or seeking employment), ii) activities done as part of travel to and from places (for work, shopping, marketing, place of worship, etc.), and iii) leisure time or recreational physical activities (sports, fitness, leisure activity, recreational, and other activities). We followed the WHO global guidelines on physical activity for adult health, categorized as vigorous activity, moderate activity and light activity or physical inactivity [11]. Vigorous activity includes individuals spending at least 75 min on a vigorously intensive activity at work, during travel, during leisure time, or in a recreational activity on a typical day. Moderate activity includes individuals spending time at least 150 min on moderately intensive activity at work, during travel, during leisure time, or in a recreational activity on a typical day. Light or physical inactivity include any activity that does not fall in the above two categories.

The SAGE questions on physical activity allow for a direct comparison with the Global Physical Activity Questionnaire (GPAQ) survey [32]. The Global Physical Activity Questionnaire was developed by the WHO to measure physical activity across the work, travel, and recreational domains separately, and it is used in many countries as part of WHO STEP wise approach for non-communicable diseases surveillance [17].

Information on nutritional intake was obtained using two questions that asked the respondents to describe how many separate servings of fruits and vegetables they took on a typical day. Less than 5 servings of fruits and vegetables/day was coded as “1” and defined as “inadequate intake of fruit and vegetables,” and more than or equal to 5 servings of fruits and vegetables/day was coded as “2” and defined as “adequate intake of fruit and vegetables” in the analysis. For tobacco use, the survey asked the respondents whether they had ever smoked tobacco or used smokeless tobacco and whether they used any tobacco products daily. For alcohol consumption, the respondents were asked whether they had ever consumed alcohol or if they had consumed a drink that contained alcohol (such as beer, wine, spirits, etc.). The responses were coded into “0” for no and “1” for yes. The respondents were then asked how frequently (how many days) on an average they had had at least one alcoholic drink in the previous 12 months. The responses were coded as “0” for “no days”, “1” for “less than once a month”, “2” for “one to three days per month”, “3” for “one to four days per week”, and “4” for “five or more days per week”. Another question posed to the respondents was: “In the last 12 months, on the days you drank alcoholic beverages, how many drinks did you have on average?” The previous two questions were used to categorize the respondents as non-drinkers, infrequent moderate drinkers (1–3 days/month with < 2 drinks on the day), and frequent heavy drinkers (1–5 or more days/week with ≥3 drinks). Volume and pattern of drinking determine the health status of a person [14]. Therefore, we included the pattern and intensity of alcohol drinking (non-drinkers, infrequent moderate drinkers, and frequent heavy drinkers) in our study. All the information collected was analyzed to assess the behavioral habits of the respondents and to determine if those habits posed as significant risk factors for chronic diseases.

Body mass index (BMI) was also included as an exposure variable because it is one of the metabolic risk factors of chronic diseases. BMI was calculated as weight in kilograms divided by the square of the height in meters (kg/m^2^) in accordance with the WHO guidelines, and was categorized as underweight (< 18.5 kg/m^2^), normal weight (18.5–24.9 kg/m^2^), overweight (25.0–29.9 kg/m^2^), and obese (≥30 kg/m^2^).

Socioeconomic-demographic characteristics:

The socioeconomic-demographic variables included were: place of residence (rural, urban), sex (male, female), age group (18–29, 30–39, 40–49, 50–59, 60–69), and the highest level of education (no education, <primary and primary, secondary, high school and above). The wealth index was computed based on the permanent income of the respondents and categorized as poorest, poor, middle, rich, and richest. Caste group (Scheduled Caste (SC)/Scheduled Tribe (ST), other than Scheduled Caste/Scheduled Tribe), religion (Hindu, other), religious services (never or once or twice/year/month, once or twice a week/daily) were the other variables considered for the analysis.

#### Statistical analysis

The prevalence of self-reported chronic diseases was calculated as the ratio of those having the diseases divided by the total surveyed sample of the study population. The prevalence of chronic diseases was defined in terms of respondents suffering from at least one of the self-reported chronic diseases. The prevalence of symptom or measured chronic diseases was calculated and defined likewise. The bi-variable analysis was carried out with socioeconomic-demographic factors associated with BRFs. The odds of the self-reported and symptom or measured chronic diseases were computed by using the logistic regression model. The adjusted odds ratio was computed separately for all self-reported and symptom or measured chronic diseases by behavioral risk factors and socioeconomic-demographic characteristics. The multi-collinearity was tested before applying the logistic regression model. National population weight was applied in the process of data analysis. The SAGE data provides household weights for household-level analysis. Individual weights for person-level analysis were based on the selection probability at each stage of respondent selection. For our analysis, we used individual weights. Individual weights were post-stratified by the six states, locality, sex, and age groups according to the 2006 projected population estimates. All the analysis was done using Stata-13 statistical software.

## Results

Table 1 presents the results of behavioral risk factors by socioeconomic-demographic characteristics. The result found that 94.7% of the population had an inadequate intake of fruits and vegetables. Nearly half of the population used tobacco products (43.4%), about 17.0% consumed alcohol, and 21.6% was physically inactive. The number of adults who reported alcohol consumption, tobacco use, and inadequate intake of fruits and vegetables was more in the rural areas, whereas the number of physically inactive adults was more in the urban areas. Females were more physically active, reported more inadequate intake of fruits and vegetables, and were less engaged in alcohol and tobacco consumption. Alcohol consumption was higher in the age group of 30–44 years (21.8%), and tobacco use was more in the 60–69 years age group (56.5%). Physical inactivity was found to increase with an increase in age and was higher among the high school and above educational attainment group. Obese and overweight adults were more physically inactive; obese and underweight adults had an inadequate intake of fruit and vegetables, and underweight adults were highly engaged in alcohol and tobacco use. Physical inactivity was more in the richest quintile, whereas tobacco and alcohol consumption was more in the poorest quintile.

**Table 1:** Behavioral risk factors (BRFs) by socioeconomic-demographic characteristics among adult population (18–69 years) in India, 2007

Socioeconomic-demographic characteristics	Physical inactivity	Inadequate intake of fruits and vegetables^#^	Alcohol consumption^**^**^	Any tobacco use	Surveyed sample (*N*)
Place of residence					
Urban	23.7	93.0	13.9	35.3	2481
Rural	20.6	95.5	18.4	47.1	7358
Household’s religion					
Hindu	21.1	94.8	18.6	42.8	8243
Others^+^	24.0	94.4	8.9	47.0	1596
Household’s caste group					
Scheduled Caste (SC) and Scheduled Tribe (ST)	18.8	96.5	25.9	50.5	2490
Other than SC/ST^++^	22.5	94.2	14.1	41.1	7349
Religious services					
Never or once or twice/year/month	21.3	94.8	17.2	42.9	9279
Once or twice/week/daily	25.1	93.3	13.4	51.9	560
Member’s sex					
Male	23.1	92.8	31.5	65.5	3585
Female	19.9	96.8	1.5	19.9	6254
Age group					
18–29	23.3	95.1	8.6	25.1	1611
30–44	17.1	95.1	21.8	46.2	2403
45–59	22.8	93.5	18.5	54.0	3598
60–69	34.9	95.3	15.3	56.5	2227
Educational attainment^+++^					
No education	19.4	97.9	15.2	46.6	4223
<primary & primary	20.3	96.4	19.5	47.6	2558
Secondary	21.6	93.0	18.4	41.8	1296
High school & above	25.6	90.1	15.9	36.2	1762
Body mass Index (BMI)					
Underweight	21.5	96.5	18.2	51.1	3212
Normal weight	20.9	94.0	17.2	41.8	5027
Overweight	23.9	92.0	15.4	31.3	1064
Obese	26.1	96.5	8.1	30.2	362
Wealth Quintile					
Poorest	19.7	98.3	19.1	56.0	1607
Poor	17.2	96.2	18.9	47.5	1854
Middle	18.6	95.3	17.3	47.4	1925
Rich	23.1	93.3	13.7	38.5	2260
Richest	29.3	90.5	15.9	27.5	2193
Total	21.6	94.7	17.0	43.4	9839

^#^ The inadequate intake of fruits and vegetables includes < 5 serving of fruits and vegetables/ day

^+^ Other religion includes Islam, Jainism, Buddhism, Sikhism, Christian and other religion

^++^ Other than SC/ST includes Other Backward Caste (OBC) and other caste

^+++^ Educational attainments present the highest level of education completed by members

^**^**^ Alcohol consumption included ever or current drinkers that drink contain alcohol such as such as beer, wine, spirits, etc.

Note: Percentage distributions presented in the table are weighted analysis, and presented surveyed sample (N) are un-weighted

The prevalence of the self-reported chronic diseases by BRFs and socioeconomic-demographic covariates result is presented in Table 2. The prevalence of self-reported chronic diseases was 24.1%, and it was higher among urban residents than their rural counterparts. The disease prevalence varied for the young versus the old age, no education versus higher education, poorest versus richest, and normal weight versus overweight population. The prevalence of chronic diseases was higher among those who had BRFs of chronic diseases. For example, the prevalence of self-reported chronic diseases was higher among those who were physically inactive (28.6%), had an inadequate intake of fruits and vegetables (24.1%), and engaged in alcohol (26.2%) and tobacco consumption (26.3%) as compared to the adults with a comparatively low level of BRFs. The significant differences in the prevalence of self-reported chronic diseases by BRFs and socioeconomic and demographic characteristics are presented in (Additional file 2: Table A2).

**Table 2:** Self-reported prevalence of chronic diseases by behavioral risk factors and socioeconomic-demographic characteristics among adult population (18–69 years) in India, 2007

Socioeconomic-demographic characteristics	Prevalence of self-reported chronic diseases	Physical activity		Nutrition		Alcohol consumption^^^		Any tobacco use	
		Physical inactivity	Vigorous activity	Inadequate intake of fruits and vegetables^#^	Adequate intake of fruits and vegetables ^##^	Yes	No	Yes	No
Place of residence									
Urban	25.3	29.7	22.3	25.6	20.5	26.1	25.1	28.7	23.4
Rural	23.5	28.1	20.5	23.5	24.4	26.2	22.9	25.5	21.7
Household’s religion									
Hindu	24.0	28.7	21.3	24.0	24.9	25.6	23.7	26.4	22.2
Others^+^	24.3	28.3	19.1	25.0	12.5	32.1	23.5	25.7	23.1
Household’s caste group									
Scheduled Caste (SC) and Scheduled Tribe (ST)	19.4	23.1	16.9	19.7	11.3	20.8	19.0	22.3	16.5
Other than SC/ST^++^	25.6	30.2	22.5	25.6	25.1	29.5	25.0	27.9	24.0
Religious services									
Never or once or twice/year/month	23.4	27.8	20.3	23.4	23.4	25.3	23.0	25.7	21.7
Once or twice/week/daily	34.4	40.1	30.1	35.8	15.3	44.5	32.9	34.3	34.6
Member’s sex									
Male	23.7	30.9	19.6	24.0	20.6	26.2	22.6	25.2	21.0
Female	24.4	25.8	23.2	24.3	28.1	26.2	24.4	30.3	23.0
Age group									
18–29	9.6	13.5	7.3	9.7	7.2	16.2	9.0	12.5	8.6
30–44	23.4	28.6	21.1	23.5	22.2	22.4	23.7	23.8	23.0
45–59	34.2	35.7	32.4	34.4	30.8	33.8	34.2	31.1	37.8
60–69	43.5	47.5	37.2	43.5	44.7	43.5	43.5	42.5	44.9
Educational attainment^+++^									
No education	25.3	30.9	21.7	25.3	27.0	23.8	25.6	25.4	25.2
<primary & primary	24.0	28.4	21.3	23.6	32.6	23.0	24.2	25.0	23.0
Secondary	23.3	27.6	19.4	24.2	12.2	28.1	22.2	28.4	19.7
High school & above	23.1	27.1	20.6	23.1	23.0	31.7	21.4	27.9	20.3
Body mass Index (BMI)									
Underweight	20.8	27.9	20.1	20.9	18.5	24.9	19.9	23.4	18.1
Normal weight	23.0	26.7	16.6	23.1	21.1	24.5	22.6	25.4	21.2
Overweight	39.9	39.1	41.1	40.6	32.5	38.3	40.2	47.0	36.7
Obese	29.5	27.3	31.2	28.9	47.3	46.5	28.1	34.1	27.6
Wealth Quintile									
Poorest	20.8	24.6	19.6	20.9	11.1	18.6	21.3	22.9	18.1
Poor	21.6	33.4	16.7	21.5	25.5	23.9	21.1	24.7	18.8
Middle	25.8	26.9	24.2	26.6	10.0	25.0	26.0	28.2	23.6
Rich	24.3	31.2	19.9	24.2	25.1	28.4	23.6	26.5	22.9
Richest	27.9	27.7	25.5	27.9	28.3	37.5	26.1	32.6	26.2
Total	24.1	28.6	20.9	24.1	22.8	26.2	23.6	26.3	22.3

^#^ Inadequate intake of fruit and vegetables includes < 5 serving of fruit and vegetables/ day

^##^ Adequate intake of fruit and vegetables includes ≥5 serving of fruit and vegetables/ day

^+ ++ +++^Same define as in the Table 1

^**^**^Alcohol consumption included ever or current drinkers that drink contain alcohol such as beer, wine, spirits, etc.

Note: Diseases prevalence (in %) presented in the table are weighted analysis

Table 3 presents the prevalence of symptom or measured chronic diseases by behavioral risk factors and socioeconomic-demographic characteristics. The prevalence of symptom or measured chronic diseases was 41.9%, which was higher in females. The prevalence increased with increased age and decreased with increases in the level of education of members. It was also higher among physically inactive (39.4%) adults of urban areas and among the alcohol and tobacco users (46.9% versus 45.4%) from rural areas. The highly educated population of those who consumed alcohol and tobacco products also showed a higher prevalence. Similar results were found among the overweight, obese, and the richest population groups. The significant differences in prevalence of symptom based or measured chronic diseases by BRFs and socioeconomic and demographic characteristics are presented in (Additional file 3: Table A3).

**Table 3:** Symptom or measured prevalence of chronic diseases by behavioral risk factors and socioeconomic-demographic characteristics among adult population (18–69 years) in India, 2007

Socioeconomic-demographic characteristics	Prevalence of symptom or measured chronic diseases	Physical activity		Nutrition		Alcohol consumption^^^		Any tobacco use	
		Physical inactivity	Vigorous activity	Inadequate intake of fruits and vegetables^#^	Adequate intake of fruits and vegetables ^##^	Yes	No	Yes	No
Place of residence									
Urban	40.7	39.4	39.4	41.1	35.4	44.7	40.1	40.9	40.6
Rural	42.4	36.3	42.2	42.4	43.2	46.9	41.4	45.4	39.7
Household’s religion									
Hindu	41.7	37.2	41.9	41.8	40.4	46.3	40.7	44.4	39.7
Others^+^	42.7	38.1	38.8	43.0	37.8	46.6	42.4	43.6	42.0
Household’s caste group									
Scheduled Caste (SC) and Scheduled Tribe (ST)	42.1	34.3	41.7	42.3	36.1	48.7	39.7	47.4	36.6
Other than SC/ST^++^	41.8	38.2	41.4	41.9	40.7	44.9	41.3	43.0	41.0
Religious services									
Never or once or twice/year/month	41.9	38.0	41.5	42.0	40.6	46.2	41.1	45.4	39.3
`Once or twice/week/daily	40.8	28.6	40.9	41.4	32.8	48.5	39.7	29.6	52.9
Member’s sex									
Male	37.2	33.5	37.2	37.3	35.3	46.0	33.1	41.4	29.2
Female	46.9	42.2	48.3	46.8	51.2	54.5	46.8	54.6	45.0
Age group									
18–29	23.0	13.5	28.2	23.2	19.6	23.5	23.0	28.1	21.4
30–44	41.9	38.1	41.2	42.1	37.8	43.7	41.4	40.2	43.3
45–59	53.8	48.4	51.8	53.8	53.1	57.3	53.0	50.8	57.2
60–69	66.6	65.4	66.7	66.7	64.5	66.0	66.7	65.6	67.9
Educational attainment^+++^									
No education	49.3	45.8	46.6	49.1	58.8	43.1	50.4	48.3	50.2
<primary & primary	45.2	47.9	42.7	45.1	48.4	50.6	43.9	44.5	45.8
Secondary	35.9	24.1	39.5	35.0	47.3	48.1	33.1	42.7	31.0
High school & above	33.0	28.0	34.3	33.5	28.4	43.6	30.9	38.6	29.8
Body mass Index (BMI)									
Underweight	39.8	36.0	38.6	40.9	45.7	45.7	40.1	44.5	37.5
Normal weight	41.1	36.7	41.0	40.0	36.6	42.3	39.3	40.0	39.6
Overweight	56.5	49.6	60.4	57.8	42.2	67.3	54.6	69.8	50.5
Obese	42.4	30.5	47.1	41.7	61.2	83.1	38.8	59.8	34.8
Wealth Quintile									
Poorest	42.5	43.5	40.6	42.4	47.8	46.2	41.7	42.4	42.6
Poor	42.9	39.9	43.4	42.4	54.9	46.7	42.0	43.5	42.3
Middle	43.0	36.1	42.8	43.6	31.2	36.3	44.4	44.8	41.4
Rich	42.7	42.4	41.8	42.4	46.3	50.2	41.5	45.6	40.8
Richest	38.3	28.6	37.9	38.9	32.4	53.6	35.4	46.8	35.1
Total	41.9	37.4	41.5	42.0	40.0	46.3	41.0	44.3	40.0

^# ## + ++ +++^ Same define as in Table 1 and Table 2

^**^**^ Alcohol consumption included ever or current drinkers that drink contain alcohol such as such as beer, wine, spirits, etc.

Note: Diseases prevalence (in %) presented in the table are weighted analysis

The self-reported and symptom-based or measured chronic diseases examined in the logistic regression model are presented in Tables 4 and 5. The regression models were run separately for each chronic disease. Table 4 results show that moderate physical activity was significantly less likely to be associated with self-reported depression, arthritis, and stroke, while vigorous activity was more likely to be associated with depression, COPD, and arthritis than physical inactivity. Adequate intake of fruits and vegetables was significantly less likely to be associated with angina (OR: 0.31; 95% CI: 0.16–0.60), COPD (OR: 0.22; 95% CI: 0.09–0.54), and asthma (OR: 0.20; 95% CI: 0.10–0.42), but more likely to be associated with depression (OR: 3.45; 95% CI: 1.99–5.97). Infrequent moderate and frequent heavy alcohol drinking were less likely to be associated with asthma and angina than non-drinking. Consumers of any tobacco product were four times more likely to have stroke (OR: 3.59; 95% CI: 1.07–12.13) compared to non-tobacco users. Overweight adults were significantly more likely to have hypertension, arthritis, and diabetes than normal weight adults. Likewise, obese adults were significantly more likely to have hypertension (OR: 2.30; 95% CI: 1.49–3.53). Underweight adults, on the other hand, were more likely to have COPD (OR: 2.52; 95% CI: 1.57–4.04) than normal weight adults. Females were significantly more likely to report hypertension (OR: 2.32 95% CI: 1.70–3.17) and arthritis (OR: 1.70; 95% CI: 1.27–2.27), but less likely to be report COPD (OR: 0.37; 95% CI: 0.21–0.65) and diabetes (OR: 0.40; 95% CI: 0.24–0.66) than males. Increased age was a significant factor of self-reported chronic diseases (Table 4).

**Table 4:** Odds ratio of self-reported chronic diseases by behavioral risk factors and socioeconomic-demographic characteristics among adult population (18–69 years) in India, 2007

Socioeconomic-demographic characteristics	Self-reported chronic diseases (Adjusted odds ratio, 95% CIs)							
	Angina	Depression	Hypertension	COPD	Arthritis	Asthma	Stroke	Diabetes
Physical activity								
Physical inactivity^@^								
Moderate active	1.28(0.77,2.13)	0.30*(0.18,0.50)	1.09(0.80,1.48)	0.74(0.42,1.29)	0.68*(0.52,0.90)	1.24(0.74,2.09)	0.22*(0.09,0.54)	1.36(0.76,2.45)
Vigorous active	0.90(0.50,1.61)	0.47*(0.28,0.78)	1.02(0.73,1.44)	0.36*(0.20,0.66)	0.73*(0.55,0.99)	1.04(0.61,1.78)	0.49(0.22,1.12)	0.94(0.54,1.62)
Nutritional status								
Inadequate intake^# @^								
Adequate intake^##^	0.31*(0.16,0.60)	3.45*(1.99,5.97)	0.76(0.48,1.20)	0.22*(0.09,0.54)	1.24(0.78,1.96)	0.20*(0.10,0.42)	0.77(0.20,2.96)	0.80(0.36,1.81)
Alcohol consumption^^^								
Non-drinkers^@^								
Infrequent moderate drinkers	0.38 (0.15,1.01)	0.98(0.48,1.99)	1.63(0.88,3.03)	1.53(0.62,3.77)	1.12(0.55,2.28)	0.38*(0.19,0.77)	1.29(0.23,7.20)	0.57(0.23,1.40)
Frequent heavy drinkers	0.13*(0.03,0.59)	1.72(0.61,4.80)	0.93(0.22,4.01)	0.32(0.10,1.00)	1.84(0.65,5.22)	1.37(0.51,3.73)	0.26(0.05,1.41)	1.49(0.24,9.12)
Any tobacco use								
No^@^								
Yes	1.31(0.84,2.05)	1.07(0.67,1.72)	1.17(0.86,1.59)	1.06(0.58,1.92)	1.06(0.80,1.40)	0.91(0.57,1.46)	3.59*(1.07,12.13)	0.93(0.58,1.47)
Body mass Index								
Normal weight^@^								
Underweight	0.79(0.49,1.27)	0.84(0.54,1.32)	0.82(0.60,1.11)	2.52*(1.57,4.04)	0.67*(0.52,0.87)	1.41(0.87,2.27)	0.33*(0.15,0.73)	0.94(0.49,1.82)
Overweight	1.02(0.58,1.77)	1.83(0.92,3.63)	1.91*(1.32,2.75)	0.28*(0.14,0.55)	2.35*(1.68,3.28)	1.29(0.55,3.03)	0.84(0.27,2.59)	2.79*(1.55,5.01)
Obese	0.80(0.32,1.98)	0.74(0.32,1.74)	2.30*(1.49,3.53)	0.93(0.28,3.03)	0.87(0.51,1.49)	1.82(0.60,5.58)	1.66(0.29,9.61)	1.70(0.95,3.05)
Place of residence								
Urban^@^								
Rural	0.78(0.45,1.35)	1.22(0.77,1.95)	0.85(0.64,1.12)	1.74(0.94,3.23)	1.45*(1.08,1.96)	0.82(0.52,1.32)	0.66(0.26,1.71)	0.88(0.53,1.45)
Member’s sex								
Male^@^								
Female	1.04(0.71,1.52)	0.58(0.25,1.35)	2.32*(1.70,3.17)	0.37*(0.21,0.65)	1.70*(1.27,2.27)	0.59(0.34,1.03)	1.53(0.64,3.69)	0.40*(0.24,0.66)
Age group								
18-29^@^								
30–44	9.12*(4.05,20.56)	3.03*(1.43,6.42)	3.11*(1.89,5.12)	2.79*(1.17,6.67)	2.93*(1.83,4.72)	2.38(0.93,6.10)	0.70(0.16,3.00)	2.92*(0.94,9.07)
45–59	7.24*(3.28,15.98)	3.44*(1.62,7.29)	6.52*(3.93,10.83)	3.63*(1.62,8.12)	5.98*(3.75,9.54)	3.48*(1.34,9.00)	0.99(0.21,4.79)	5.94*(1.94,18.2)
60–69	11.84*(5.08,27.61)	3.22*(1.46,7.10)	7.80*(4.60,13.23)	3.97*(1.74,9.05)	8.89*(5.53,14.28)	7.53*(3.01,18.86)	1.76(0.31,9.95)	7.91*(2.54,24.59)
Educational attainment^+++^								
No education^@^								
<primary & primary	0.95(0.59,1.53)	1.17(0.72,1.89)	0.93(0.70,1.25)	1.10(0.62,1.93)	1.15(0.88,1.51)	0.80(0.50,1.29)	1.53(0.71,3.28)	1.00(0.58,1.71)
Secondary	2.47*(1.31,4.66)	1.06(0.50,2.23)	1.28(0.86,1.91)	1.66(0.79,3.47)	1.32(0.91,1.93)	0.92(0.45,1.87)	0.99(0.18,5.31)	1.10(0.50,2.41)
High school & above	1.25(0.58,2.70)	1.09(0.57,2.07)	1.48(0.97,2.25)	1.33(0.60,2.93)	0.84(0.53,1.34)	1.03(0.54,1.96)	0.61(0.20,1.83)	1.04(0.49,2.22)
Wealth Quintile								
Poorest^@^								
Poor	0.83(0.40,1.68)	1.16(0.60,2.24)	1.14(0.72,1.81)	0.62(0.32,1.19)	0.90(0.62,1.30)	0.99(0.56,1.77)	2.75(0.86,8.74)	0.90(0.37,2.21)
Middle	1.24(0.58,2.64)	1.13(0.60,2.12)	1.71*(1.09,2.69)	0.66(0.33,1.34)	0.97(0.66,1.43)	0.68(0.36,1.27)	1.53(0.50,4.70)	2.03(0.77,5.33)
Rich	0.89(0.42,1.92)	1.46(0.78,2.75)	1.75*(1.10,2.80)	0.78(0.39,1.58)	0.70(0.47,1.06)	1.03(0.52,2.01)	1.08(0.27,4.24)	1.30(0.51,3.32)
Richest	0.71(0.35,1.47)	0.69(0.33,1.43)	2.09*(1.28,3.42)	0.39(0.15,1.06)	0.58*(0.38,0.87)	1.12(0.46,2.72)	4.47*(1.06,18.88)	2.12(0.81,5.54)

Significant at * *p* < 0.05, ^@^ reference category

Other control variables are place of residence, religion, caste, and religious services

^# ## +++^ same define as in Table 1

^^^ Infrequent moderate alcohol drinkers includes 1–3 days/month with < 2 drinks, and frequent heavy alcohol drinkers includes 1–5 or more days/week with ≥3 drinks

Note: Odds ratio presented in table are adjusted odds ratio and based on weighted analysis

**Table 5:** Odds ratio of symptom or measured chronic diseases by behavioral risk factors and socioeconomic-demographic characteristics among adult population (18–69 years) in India, 2007

Socioeconomic-demographic characteristics	Symptom or measured chronic diseases (Adjusted odds ratio, 95% CIs)							
	Angina	Depression	Hypertension	COPD	Arthritis	Asthma	Stroke	Diabetes
Physical activity								
Physical inactivity^@^								
Moderate active	1.37(0.97,1.94)	1.14(0.84,1.53)	1.20(0.89,1.63)	1.14(0.85,1.52)	1.39*(1.06,1.82)	1.75*(1.19,2.57)	0.69(0.38,1.25)	1.22(0.63,2.35)
Vigorous active	1.55*(1.08,2.22)	1.04(0.77,1.40)	1.09(0.79,1.50)	1.38*(1.05,1.81)	1.25(0.95,1.65)	1.88*(1.30,2.73)	0.63(0.35,1.14)	0.76(0.41,1.41)
Nutritional status								
Inadequate intake^# @^								
Adequate intake^##^	1.29(0.66,2.55)	0.90(0.53,1.51)	1.07(0.63,1.81)	0.54*(0.30,0.98)	1.16(0.76,1.77)	0.58(0.25,1.35)	1.43(0.75,2.72)	0.47(0.22,1.03)
Alcohol consumption^^^								
Non-drinkers^@^								
Infrequent moderate drinkers	1.23(0.63,2.42)	1.30(0.71,2.36)	1.57(0.91,2.71)	1.83*(1.04,3.21)	0.94(0.56,1.58)	0.87(0.43,1.76)	0.90(0.24,3.38)	0.23*(0.09,0.57)
Frequent heavy drinkers	0.71(0.22,2.33)	0.41(0.15,1.12)	0.52(0.23,1.19)	2.07(0.86,4.97)	1.86(0.81,4.29)	1.02(0.40,2.62)	0.56(0.09,3.46)	1.64(0.25,10.55)
Any tobacco use								
No^@^								
Yes	1.15(0.86,1.55)	1.11(0.85,1.45)	1.19(0.89,1.59)	0.91(0.71,1.17)	1.17(0.94,1.46)	1.14(0.82,1.60)	1.57(0.91,2.69)	1.02(0.60,1.71)
Body mass Index								
Normal weight^@^								
Underweight	1.00 (0.77,1.31)	1.30(1.00,1.68)	0.50*(0.39,0.66)	0.94(0.75,1.18)	0.86(0.69,1.06)	1.40*(1.00,1.96)	0.92(0.56,1.49)	1.05(0.50,2.17)
Overweight	1.48(0.90,2.45)	1.21(0.82,1.77)	2.24*(1.57,3.19)	0.79(0.54,1.16)	2.14*(1.57,2.92)	1.40(0.77,2.54)	1.36(0.63,2.93)	2.63*(1.38,5.00)
Obese	1.08(0.62,1.86)	1.13(0.63,2.03)	2.93*(1.71,5.03)	0.50*(0.25,0.99)	1.34(0.88,2.04)	0.95(0.50,1.8)	0.15*(0.05,0.50)	1.66(0.85,3.24)
Place of residence								
Urban^@^								
Rural	1.19(0.84,1.68)	0.93(0.69,1.26)	0.78(0.59,1.03)	1.07(0.80,1.42)	1.58*(1.20,2.07)	1.34(0.9,1.99)	1.68(0.89,3.17)	0.78(0.44,1.38)
Member’s sex								
Male^@^								
Female	1.78*(1.31,2.42)	1.39*(1.03,1.88)	1.40*(1.04,1.88)	1.84*(1.37,2.48)	2.08*(1.65,2.62)	0.70(0.48,1.03)	1.62(0.85,3.07)	0.37*(0.21,0.66)
Age group								
18-29^@^								
30–44	1.63(0.99,2.67)	2.89*(1.86,4.48)	2.73*(1.68,4.43)	1.46*(1.08,1.98)	2.94*(1.98,4.36)	1.95*(1.03,3.70)	2.43(0.87,6.81)	2.95(0.78,11.09)
45–59	2.16*(1.30,3.61)	3.19*(2.06,4.95)	6.67*(4.08,10.92)	1.51*(1.10,2.05)	5.32*(3.53,8.03)	3.12*(1.63,6.00)	2.94*(1.08,8.05)	4.87*(1.29,18.41)
60–69	3.45*(2.11,5.64)	4.82*(3.09,7.52)	10.4*(6.30,17.17)	1.71*(1.22,2.39)	8.75*(5.76,13.28)	5.56*(2.92,10.6)	4.49*(1.56,12.89)	6.46*(1.69,24.71)
Educational attainment^+++^								
No education^@^								
<primary & primary	0.96(0.71,1.30)	0.79(0.61,1.04)	1.12(0.85,1.48)	1.02(0.77,1.34)	1.01(0.81,1.26)	0.81(0.56,1.16)	1.35(0.74,2.47)	0.91(0.48,1.70)
Secondary	0.79(0.51,1.21)	0.90(0.61,1.33)	0.89(0.62,1.29)	1.34(0.94,1.90)	0.81(0.57,1.14)	0.67(0.38,1.18)	1.77(0.69,4.53)	1.13(0.46,2.78)
High school & above	0.62(0.36,1.04)	0.69(0.42,1.12)	1.07(0.74,1.55)	1.11(0.72,1.71)	0.83(0.57,1.21)	0.97(0.55,1.71)	1.23(0.41,3.65)	0.79(0.33,1.87)
Wealth Quintile								
Poorest^@^								
Poor	1.00(0.67,1.48)	1.01(0.71,1.43)	0.80(0.53,1.22)	1.43(1.00,2.05)	0.72*(0.53,0.97)	1.02(0.68,1.54)	0.80(0.39,1.63)	0.90(0.29,2.80)
Middle	0.79(0.55,1.15)	1.15(0.80,1.65)	1.05(0.71,1.55)	1.56*(1.08,2.25)	0.77(0.58,1.03)	1.09(0.72,1.64)	0.63(0.30,1.30)	3.09*(1.00,9.56)
Rich	1.06(0.72,1.58)	1.01(0.68,1.51)	1.17(0.77,1.76)	1.28(0.88,1.86)	0.73(0.52,1.01)	1.03(0.67,1.58)	0.61(0.28,1.34)	2.02(0.70,5.88)
Richest	0.47*(0.29,0.75)	0.60*(0.39,0.93)	1.27(0.80,2.01)	1.12(0.74,1.70)	0.54*(0.38,0.77)	0.79(0.42,1.48)	0.50(0.21,1.17)	3.04*(1.11,8.36)

Significant at * *p* < 0.05, ^@^reference category

Other control variables are place of residence, religion, caste, and religious services

^# ## +++^ same define as in Table 1

^^^ Infrequent moderate alcohol drinkers includes 1–3 days/month with < 2 drinks, and frequent heavy alcohol drinkers includes 1–5 or more days/week with ≥3 drinks

Note: Odds ratio presented in table are adjusted odds ratio and based on weighted analysis

The results of the logistic regression model of the symptom or measured chronic diseases by BRFs and socioeconomic-demographic characteristics are presented in Table 5. Vigorous physical activity was significantly more likely to be associated with the symptom or measured based angina (OR: 1.55; 95% CI: 1.08–2.22), COPD (OR: 1.38; 95% CI: 1.05–1.81), and asthma (OR: 1.88; 95% CI: 1.30–2.73) than physical inactivity. Similarly, moderate activity was significantly more likely to be associated with asthma (OR: 1.75; 95% CI: 1.19–2.57) and arthritis (OR: 1.39; 95% CI: 1.06–1.82) than physical inactivity. Adequate intake of fruits and vegetables was significantly less likely to be associated with COPD (OR: 0.54; 95% CI: 0.30–0.98) than the inadequate intake of fruits and vegetables. Infrequent moderate alcohol drinkers were statistically more likely to report COPD (OR: 1.83; 95% CI: 1.04–3.21), but less likely to report diabetes (OR: 0.23; 95% CI: 0.09–0.57) than non-drinkers. The tobacco users were not significantly associated with the symptom or measured based chronic diseases. The higher BMI adults were significantly more likely to be associated with hypertension than normal weight adults. Similarly, overweight adults were two times more likely to be associated with arthritis (OR: 2.14; 95% CI: 1.57–2.92) and three times more likely to be associated with diabetes (OR: 2.63; 95% CI: 1.38–5.00). Underweight adults, on the other hand, were more likely to be associated with asthma (OR: 1.40; 95% CI: 1.00–1.96). Adults residing in rural areas reported arthritis more than those residing in urban areas. Females were found to be significantly more likely associated with angina, depression, hypertension, COPD, and arthritis; however, they were less likely to be associated with diabetes compared to the male population. Increased age was a significant predictor of the symptom or measured chronic diseases.

## Discussion

In the present cross-sectional study, we assessed the association of behavioral risk factors with self-reported and symptom or measured chronic diseases in India. Our results indicate that there was a higher prevalence of symptom or measured chronic diseases than that of self-reported chronic diseases. A study conducted in low middle-income countries has also shown that symptom-based or measured chronic non-communicable diseases have a higher prevalence than self-reported chronic non-communicable diseases [26]. Our study found that vigorous physical activity was significantly less likely to be associated with self-reported chronic diseases such as COPD, depression, and arthritis. Likewise, moderate activity too was less likely to be associated with depression, arthritis, and stroke. This finding is similar to that of another study, which found that regular physical activity was related to lower blood pressure in young and older adults [12]. Physical activity helps in weight loss or reduction in total fat, which ultimately helps to reduce blood pressure and to control blood glucose by increasing the insulin sensitivity, thereby preventing Type 2 diabetes [12, 33]. Physical activity has been found to act as primary prevention against 35 chronic conditions such as metabolic syndrome, insulin resistance, pre-diabetes, non-alcoholic fatty liver, CHD, hypertension, stroke, depression, anxiety, etc. [34]. However, compared to self-reported diseases, vigorous activity was significantly more likely to be associated with the symptom or measured chronic diseases of COPD, asthma, and angina.

The present study findings indicate that an adequate intake of fruits and vegetables is less likely to be associated with angina, asthma, and COPD. The assessment of symptom-based and measured chronic diseases also found that an adequate intake of fruits and vegetables protects people from COPD. However, the study also highlights that depression was more likely to be associated with an adequate intake of fruits and vegetables. Our study is consistent with another study that found that intake of fruits and vegetables might be inversely associated with depression [35]. However, a systematic review and meta-analysis found that the intake of fruits and vegetables was positively associated with depression [36]. For every 100 g increase in the intake of fruits and vegetables, there is a 3% decrease in the risk of depression [36]. Our findings indicate that infrequent moderate alcohol consumption is less likely to be associated with asthma and diabetes, whereas frequent heavy drinking is associated with angina. There is a significant association of moderate alcohol drinking with COPD. Tobacco use is a significant factor of stroke.

Both self-reported and symptom-based or measured chronic diseases of hypertension, arthritis, and diabetes are associated with overweight and obesity. Underweight individuals are more likely to have COPD and asthma. A micro-level study conducted in Bihar found that alcohol consumption leads to weight gain, which is one of the portent risks of chronic diseases [37]. Traversy and Chaput (2015) found in their study that recreational alcohol consumption may lead to weight gain if the physical activity does not compensate it [38]. Contrary to this result, another study found an insignificant effect of moderate alcohol consumption on ischemic heart disease [39]. However, the protective effects of light alcohol consumption, through increased HDL cholesterol, lower plasma fibrinogen, and inhibited platelet aggregation, may not counterbalance the adverse effects of high blood pressure and triglycerides on cardiovascular diseases [39]. A study by Roy et al. (2010) in India found that protective physiologies like total LDL and HDL cholesterol, BMI, systolic and diastolic blood pressure, and fasting blood sugar were higher among alcohol users compared to lifetime abstainers [40].

The current study found that both self-reported and symptom or measured chronic diseases such as hypertension, arthritis, angina, depression, and COPD were significantly associated with the female population. Females were less physically active, had an inadequate intake of fruits and vegetables, and were less engaged in alcohol and tobacco use than males. Our findings are consistent with other studies, which have found that females spend more time in accumulated household work and sedentary activities. They are busy taking care of their families and children and pay less attention to themselves [41]. Alcohol and tobacco consumption are less prevalent in females because drinking alcohol and smoking tobacco may not be socially acceptable for them. By contrast, alcohol drinking is considered a social activity among males and is accepted as a form of casual behavior [42]. Our study also found that the urban populations were less physically active than the rural populations. This disparity could be due to the less physically demanding nature of the jobs in the urban areas and the availability of mechanized transport and household appliances among urban dwellers [43].

The study findings show that age was an essential predictor of both self-reported and symptom or measured chronic diseases. Adequate intake of fruits and vegetables was found to have elevated odds but was not statistically associated with the symptom or measured chronic diseases of angina, hypertension, arthritis, and stroke. Similarly, moderate and vigorous activity were found to have elevated odds of angina, COPD, and arthritis (symptom or measured). There may be some other factors too that determine health status. Other studies have revealed that consumption of high-energy foods and participation in little physical activity are results of abdominal obesity [21, 37]. A study by Mishra et al. (2009) found that overweight/obesity were significant drivers of increased metabolic syndrome and diabetes. Around 10–15% of the Indian population is believed to be overweight/obese, and this situation requires appropriate management for prevention from diseases [44].

### Limitations

This study has several strengths and weaknesses. One of its significant strengths is that it used a nationally representative population-based survey data to examine the behavioral risk factors and their association with self-reported and symptom or measured chronic diseases in India. A key limitation of this study is that it could not infer the causal relationship due to the cross-sectional study design. For instance, the data on nutrition focused on the number of servings of fruits and vegetables in a typical day, but the serving size was unclear. Some previous studies have suggested serving sizes of fruits and vegetables that are below (90.9% population consumed less than 400 g/day) the WHO/FAO recommendation levels [13, 45, 46]. Other studies have recommended more than five servings of fruits and vegetables [13]. However, due to the cross-sectional study design and the unavailability of the second round of the SAGE data, this study could not establish whether the intake of fruits and vegetables increased before or after the onset of diseases. For establishing causal relationships, there needs to be a longitudinal study.

Another limitation pertains to the onset of physical activities. The study gathered information on the intensity and duration of physical activity during the preceding seven days, but it did not inquire if physical activity increased before or after having contracted a disease. Yet another constraint of the study findings is related to the fact that it revealed a much higher prevalence of the symptom-based or measured chronic diseases than that of the self-reported diseases. The information on some chronic diseases included in the symptom-based estimates (angina, depression, arthritis, asthma, and stroke) may not present the accurate prevalence of the diseases. For example, using a Spirometer is the most practical and the most widely performed test for measuring the lung function and the health condition and is ideal for the early screening of the disease [47]. However, given its limited use as a screening tool, moderate COPD cannot be easily identified without the help of medical and physical examinations [48–50]. In addition, an accurate clinical diagnosis is not feasible for a large population-based survey [51]. With all these limitations, this study provides the burden of self-reported and symptom-based or measured chronic diseases. There may be a higher proportion of the population that suffers from chronic diseases but remains undiagnosed [26].

## Conclusions

The study findings reveal that the prevalence of chronic diseases and their association with BRFs and socioeconomic-demographic covariates differ markedly when assessed against self-reported criteria versus symptom or measured criteria. The adequate intake of fruits and vegetables is less likely to be associated with self-reported and symptom or measured chronic diseases. The analysis of self-reported chronic diseases also revealed that physical activity is one of the crucial preventive behaviors that control and delays the risk of chronic diseases. Moderate activity is positively associated with symptom or measured arthritis and asthma, whereas vigorous activity is positively associated with angina, COPD, and asthma. Frequent and heavy alcohol drinking is not a significant predictive factor of self-reported and symptom or measured chronic diseases. However, moderate drinking has a significant association with the symptom or measured based COPD. Tobacco consumption is positively associated with self-reported stroke. Overweight is closely associated with hypertension, COPD, arthritis, and diabetes. The study findings are consistent with what other studies have found, and it recommends that public health efforts should focus on reducing obesity and alcohol and tobacco use [52]. Overweight and physically inactive adults have a higher prevalence of chronic diseases [53]. Behavioral lifestyle interventions are likely to help mitigate the extent of the overweight epidemic [54]. In 2008, the Government of India launched the National Programme for Prevention and Control of Cancer, Diabetes, Cardiovascular Disease and Stroke (NPCDCS), with significant concern for preventing and controlling major chronic diseases and creating awareness relating to lifestyle behaviors. The present study emphasizes that the program should have a mechanism to identify those exposed to behavioral risk factors. Awareness regarding BRFs of chronic diseases and their prevention must be rigorously spread across the country through mass media and other means of communication with locally texted messages. Governments and NGOs should use strategic forums such as workplace, schools, panchayats, etc. to promote healthy behaviors and active lifestyles so that we can delay the onset of chronic diseases and reduce morbidity and mortality in the country.

## Additional file

Additional file 1: **Table A1.** Detail description of methods (DOCX 22 kb)

Additional file 2: **Table A2.** Differences in self-reported diseases by BRFs (DOCX 20 kb)

Additional file 3: **Table A3.** Differences in symptom-measured diseases by BRFs (DOCX 20 kb)

## Abbreviations

BMI: Body Mass Index
BRFs: Behavioral Risk Factors
CHD: Chronic Heart Diseases
COPD: Chronic Obstructive Pulmonary Diseases
DALY: Disability Adjusted Life Years
GPAQ: Global Physical Activity Questionnaires
HDL: High Density Lipoprotein
LDL: Low Density Lipoprotein
NPCDCS: National Programme for Prevention and Control of Cancer, Diabetes, Cardiovascular Disease and Stroke
SAGE: Study on Global Ageing and Adult Health Survey
WHO: World Health Organization
