# Supplementary material for: Association of behavioral risk factors with self-reported and symptom or measured chronic diseases among adult population (18–69 years) in India: evidence from SAGE study
Source: BMC Public Health. 2019 May 14;19:560. doi: 10.1186/s12889-019-6953-4 (PMC6518500; doi:10.1186/s12889-019-6953-4)
Supplement: Supplementary file 1 — Table A1. Detail description of methods (DOCX 22 kb) [file 12889_2019_6953_MOESM1_ESM.docx]

**Additional file 1:**

**Table A1.** Detail description of methods of self-reported and symptom or measured chronic diseases

| **Chronic diseases** | **Self-reported** | **Symptom or measurement** |
| --- | --- | --- |
| 1. Angina | Have you ever been diagnosed with angina or angina pectoris (a heart disease)?  (yes/no) | For angina, the following conditions were identified as:   1. During the last 12 months, have you experienced any pain or discomfort in your chest when you walk uphill or hurry? (response; i) yes, ii) no, iii) never walks uphill or hurries) 2. During the last 12 months, have you experienced any pain or discomfort in your chest when you walk at an ordinary pace on level ground? (yes/no) 3. What do you do if you get the pain or discomfort when you are walking? (response; i.) stop or slow down, ii) carry on after taking a pain relieving medicine that dissolves in your mouth iii) carry on walking) 4. If you stand still, what happens to the pain or discomfort? (response; i) relieved ii) not relieved). 5. Apart from these questions, respondents were asked to identify the points of pain in the upper part of the body (excluding the head) with the help of a picture.   **Symptom/measured:** If the response to questions 1 was “yes” and the response to questions 2 and 3 was the first option, and in question 4 the respondent indicated that the pain was in the upper left part of the body, the person was said to have angina. |
| 1. Depression | Have you ever been diagnosed with depression?  (yes/no) | For the depression the 18 question were asked to the respondent on the basis of symptoms as:   1. During the last 12 months, have you had a period lasting several days when you felt sad, empty, or depressed? (yes/no) 2. During the last 12 months, have you had a period lasting several days when you lost interest in most things you usually enjoy such as personal relationships, work or hobbies/recreation? (yes/no) 3. During the last 12 months, have you had a period lasting several days when you have been feeling your energy decreased or that you are tired all the time? (yes/no) 4. Was this period [of sadness/loss of interest/low energy] for more than 2 weeks? (yes/no) 5. Was this period [of sadness/loss of interest/low energy] most of the day, nearly every day? (yes/no) 6. During this period, did you lose your appetite? (yes/no) 7. Did you notice any slowing down in your thinking? (yes/no) 8. Did you notice any problems falling asleep? (yes/no) 9. Did you notice any problems waking up too early? (yes/no) 10. During this period, did you have any difficulties concentrating; for example, listening to others, working, watching TV, listening to the radio? (yes/no) 11. Did you notice any slowing down in your moving around? (yes/no) 12. During this period, did you feel anxious and worried most days (yes/no) 13. During this period, were you so restless or jittery nearly every day that you paced up and down and couldn’t sit still? (yes/no) 14. During this period, did you feel negative about yourself or like you had lost confidence? (yes/no) 15. Did you frequently feel hopeless - that there was no way to improve things? (yes/no) 16. During this period, did your interest in sex decrease? (yes/no) 17. Did you think of death, or wish you were dead? (yes/no) 18. During this period, did you ever try to end your life? (yes/no)   For depression symptom, the two sets of variable were identify as:  The first set of variables was based on questions 1–5 and question 16. From this set, 3 variables were computed taking the values 0 and 1, as follows:  1) The first variable takes the value 1 if the response to any of questions 1, 4, and 5 is “yes.”  2) The second variable takes the value 1 if the response to question 2 or 16 is “yes.”  3) The third variable takes the value 1 if the response to question 3 is “yes.”  The second set of variables was identifying on questions 6–15, 17, and 18. From these questions, 7 variables were computed.  1) The first variable takes the value 1 if the response to question 14 or 15 is “yes.”  2) The second variable takes the value 1 if the response to question 12 or 13 is “yes.”  3) The third variable takes the value 1 if the response to question 17 or 18 is “yes.”  4) The fourth variable takes the value 1 if the response to question 7 or 10 is “yes.”  5) The fifth variable takes the value 1 if the response to question 11 is “yes.”  6) The sixth variable takes the value 1 if the response to question 8 or 9 is “yes.”  7) The seventh variable takes the value 1 if the response to question 6 is “yes.”  **Symptom/measured:** These newly created variables from the respective sets were added to obtain 2 new variables, the first consisting of the sum of the first set of variables (maximum value 3) and the second consisting of the sum of the second set of variables (maximum value 7). On the basis of these 2 variables, a respondent was said to suffer from depression if s/he had a value for the first variable of 2 or more and a value for the second variable of 4 or more. |
| 1. Hypertension | Have you ever been diagnosed with high blood pressure (hypertension)?  (yes/no) | For the hypertension, blood pressure and pulse rate measurement was taken with the help of an automated recording device. The systolic and diastolic blood pressure was measured. The three seating of blood pressure reading was taken with 1 minutes of interval. The WHO classification was used and defined hypertension those having reported Diastolic Blood Pressure (DBP) ≥90 mmHg, and Systolic Blood Pressure (SBP) ≥140 mmHg. |
| 1. Chronic lung diseases (COPD) | Have you ever been diagnosed with chronic lung disease (emphysema, bronchitis, COPD)?  (yes/no) | For lung diseases test, spirometer device was used. From the respondent, it was request to take deepest breath as possible, then blowing out as hard and as fast as can and continue blowing into small tube until s/he have no breath left in the lungs. This small tube connected with spirometer device and then FEV1 (forced expiratory volume in one second) and FVC (Forced vital capacity) were documented. Then the FEV_1_ identify of the ratio of FEV1 and FVC was calculated. The Global initiative for chronic Obstructive Lung Diseases (GOLD) suggested cutoff point of mild (FEV_1_ ≥80% predicted), moderate 50%≤FEV_1_<80% predicted), severe (30%≤FEV_1_<50% predicted), and very sever (FEV_1_<30% predicted) category (GOLD report, 2018) was used. The very severe category was used for the measurement of Chronic Obstructive Pulmonary Diseases (COPD) (31). |
| 1. Arthritis | Have you ever been diagnosed with/told you have arthritis (a disease of the joints, or by other names rheumatism or osteoarthritis)?  (yes/no) | For arthritis, the symptom identified as:   1. During the last 12 months, have you experienced, pain, aching, stiffness or swelling in or around the joints (like arms, hands, legs or feet) which were not related to an injury and lasted for more than a month? (yes/no) 2. During the last 12 months, have you experienced stiffness in the joint in the morning after getting up from bed, or after a long rest of the joint without movement? (yes/no) 3. How long did this stiffness last? 1) About 30 minutes or less, 2) More than 30 Minutes 4. Did this stiffness go away after exercise or movement in the joint? (yes/no).   **Symptom/measured:** The response of question 1 and 2 was yes and 3 and 4 having first option then respondent said that have arthritis. |
| 1. Asthma | Have you ever been diagnosed with asthma (an allergic respiratory disease)?  (yes/no) | During the last 12 months, have you experienced any of the following:   1. Attacks of wheezing or whistling breathing? (yes/no) 2. Attack of wheezing that came on after you stopped exercising or some other physical activity? (yes/no) 3. A feeling of tightness in your chest? (yes/no) 4. Have you woken up with a feeling of tightness in your chest in the morning or any other time? (yes/no) 5. Have you had an attack of shortness of breath that came on without obvious cause when you were not exercising or doing some physical activity? (yes/no)   **Symptom/measured**: Respondent was suffering from asthma when s/he reported having “yes” to all subsequent questions. |
| 1. Stroke | Have you ever been told by a health professional that you have had a stroke?  (yes/no) | For stroke symptom identify as:   1. Have you ever suffered from sudden onset of paralysis or weakness in your arms or legs on one side of your body for more than 24 hours? 2. Have you ever had, for more than 24 hours, sudden onset of loss of feeling on one side of your body, without anything having happened to you immediately before? The respondent said having above symptoms reported having stroke.   **Symptom/measured:** The response of above 1 or 2 questions was yes then respondent said have stroke symptom. |
| 1. Diabetes | Have you ever been diagnosed with diabetes (high blood sugar)?  (yes/no) | For diabetes, if the respondent reported that have high blood sugar and taking insulin or other for blood sugar lowering medications in last 2 week or in 12 month said heaving diabetes. |
