# Supplementary material for: Association of behavioral risk factors with self-reported and symptom or measured chronic diseases among adult population (18–69 years) in India: evidence from SAGE study
Source: BMC Public Health. 2019 May 14;19:560. doi: 10.1186/s12889-019-6953-4 (PMC6518500; doi:10.1186/s12889-019-6953-4)
Supplement: Supplementary file 2 — Table A2. Differences in self-reported diseases by BRFs (DOCX 20 kb) [file 12889_2019_6953_MOESM2_ESM.docx]

**Additional file 2:**

**Table A2.** Differences in behavioral risk factors reported having self-reported chronic diseases by socioeconomic-demographic characteristics among adult population (18-69 years) in India, 2007

| **Socioeconomic-demographic characteristics** | **D1 (Physical inactivity-vigorous activity)** | **D2**  **(Inadequate^#^ - adequate^##^)** | **D3 (Alcohol consumers-non-consumers^^^)** | **D4 (Any tobacco users-nontobacco users)** |
| --- | --- | --- | --- | --- |
| **Place of residence** |  |  |  |  |
| Urban | 7.5* | 5.2 | 1.0* | 5.2* |
| Rural | 7.6* | -1.0* | 3.3* | 3.8* |
| **Household's religion** |  |  |  |  |
| Hindu | 7.5* | -0.9* | 2.0* | 4.2* |
| Others^+^ | 9.1* | 12.4 | 8.6 | 2.6* |
| **Household's caste group** |  |  |  |  |
| Scheduled Caste (SC) and Scheduled Tribe (ST) | 6.2* | 8.4 | 1.8 | 5.9* |
| Other than SC/ST^++^ | 7.6* | 0.5* | 4.5* | 3.9* |
| **Religious services** |  |  |  |  |
| Never or once or twice/year/month | 7.5* | 0.0* | 2.3* | 4.0* |
| Once or twice/week/daily | 10.0 | 20.5 | 11.7 | -0.3 |
| **Member's sex** |  |  |  |  |
| Male | 11.4* | 3.4 | 3.6* | 4.2 |
| Female | 2.6* | -3.8* | 1.8 | 7.3* |
| **Age group** |  |  |  |  |
| 18-29 | 6.2 | 2.5 | 7.3 | 3.9 |
| 30-44 | 7.5* | 1.3 | -1.3 | 0.8 |
| 45-59 | 3.3* | 3.6* | -0.5 | -6.7* |
| 60-69 | 10.2* | -1.2* | 0.0 | -2.4 |
| **Educational attainment^+++^** |  |  |  |  |
| No education | 9.2* | -1.8* | -1.8 | 0.2* |
| <primary & primary | 7.2* | -8.9* | -1.2 | 2.0 |
| Secondary | 8.2* | 12.0 | 5.8* | 8.7* |
| High school & above | 6.5* | 0.1* | 10.2* | 7.6* |
| **Body mass Index (BMI)** |  |  |  |  |
| Underweight | 7.8* | 2.4* | 5.1* | 5.4* |
| Normal weight | 10.2* | 1.9 | 1.8* | 4.2* |
| Overweight | -1.9* | 8.0 | -2.0* | 10.3* |
| Obese | -3.8 | -18.4* | 18.4 | 6.5 |
| **Wealth Quintile** |  |  |  |  |
| Poorest | 4.9* | 9.9 | -2.7 | 4.8* |
| Poor | 16.7* | -4.1 | 2.8 | 6.0* |
| Middle | 2.8* | 16.6 | -0.9 | 4.6* |
| Rich | 11.3* | -0.8 | 4.8* | 3.6* |
| Richest | 2.2* | -0.4* | 11.4* | 6.4 |
| **Total** | 7.7* | 1.4* | 2.6* | 4.0* |

Chi2 test significant at * p<0.05

^#^ Inadequate intake of fruits and vegetables includes <5 serving of fruits and vegetables/day

^##^  Adequate intake of fruits and vegetables includes ≥5 serving of fruits and vegetables/day

^# ## ++^ Same define as in table 1

**^^^** Alcohol consumption included ever or current drinkers that drink contain alcohol such as such as beer, wine, spirits, etc.

The differences in the prevalence based on weighted analysis.
