# Supplementary material for: Association of behavioral risk factors with self-reported and symptom or measured chronic diseases among adult population (18–69 years) in India: evidence from SAGE study
Source: BMC Public Health. 2019 May 14;19:560. doi: 10.1186/s12889-019-6953-4 (PMC6518500; doi:10.1186/s12889-019-6953-4)
Supplement: Supplementary file 3 — Table A3. Differences in symptom-measured diseases by BRFs (DOCX 20 kb) [file 12889_2019_6953_MOESM3_ESM.docx]

**Additional file 3:**

**Table A3.** Differences in behavioral risk factors reported having symptom or measured chronic diseases by socioeconomic-demographic characteristic among adult population (18-69 years) in India, 2007

| **Socioeconomic-demographic**  **characteristic** | **D1 (Physical inactivity-vigorous activity)** | **D2 (Inadequate^#^ - adequate^##^)** | **D3 (Alcohol consumer-non-consumer^^^)** | **D4 (Any tobacco user-nontobacco users)** |
| --- | --- | --- | --- | --- |
| **Place of residence** |  |  |  |  |
| Urban | 0.0 | 5.8 | 4.6 | 0.3* |
| Rural | -5.9* | -0.8* | 5.5* | 5.8* |
| **Household's religion** |  |  |  |  |
| Hindu | -4.7 | 1.4* | 5.6* | 4.8* |
| Others^+^ | -0.7* | 5.2 | 4.2 | 1.6 |
| **Household's caste group** |  |  |  |  |
| Scheduled Caste (SC) and Scheduled Tribe (ST) | -7.4 | 6.2 | 9.0* | 10.8* |
| Other than SC/ST^++^ | -3.2* | 1.1* | 3.6* | 2.1* |
| **Religious services** |  |  |  |  |
| Never or once or twice/year/month | -3.5* | 1.5* | 5.2* | 6.1* |
| Once or twice/week/daily | -12.3 | 8.6 | 8.8 | -23.3 |
| **Member's sex** |  |  |  |  |
| Male | -3.8 | 2.0 | 12.8 | 12.2* |
| Female | -6.1 | -4.4* | 7.7 | 9.6* |
| **Age group** |  |  |  |  |
| 18-29 | -14.7* | 3.6 | 0.6 | 6.7* |
| 30-44 | -3.1 | 4.3 | 2.4 | -3.1 |
| 45-59 | -3.3* | 0.7 | 4.3 | -6.4 |
| 60-69 | -1.3 | 2.2* | -0.7 | -2.3 |
| **Educational attainment^+++^** |  |  |  |  |
| No education | -0.8* | -9.7* | -7.3 | -1.9* |
| <primary & primary | 5.2 | -3.3* | 6.7* | -1.3* |
| Secondary | -15.4 | -12.3 | 15.0* | 11.8* |
| High school & above | -6.3 | 5.1 | 12.6* | 8.8* |
| **Body mass Index (BMI)** |  |  |  |  |
| Underweight | -2.6* | -4.8* | 5.7* | 7.1* |
| Normal weight | -4.3 | 3.4 | 3.0* | 0.4* |
| Overweight | -10.8 | 15.6 | 12.8* | 19.3* |
| Obese | -16.6 | -19.4 | 44.3 | 25.0* |
| **Wealth Quintile** |  |  |  |  |
| Poorest | 2.9 | -5.3* | 4.5 | -0.2* |
| Poor | -3.5 | -12.5 | 4.6 | 1.2* |
| Middle | -6.7 | 12.4 | -8.2 | 3.4* |
| Rich | 0.6 | -3.9 | 8.7* | 4.8* |
| Richest | -9.3 | 6.5 | 18.2* | 11.7* |
| **Total** | **-4.1*** | **2.0*** | **5.4*** | **4.3*** |

Chi2 test significant at * p<0.05

^#^  Inadequate intake of fruit and vegetables includes <5 serving of fruit and vegetables/day

^##^ Adequate intake of fruit and vegetables includes ≥5 serving of fruit and vegetables/day

^# ## + ++ +++^ Same define as in table 1

**^^^** Alcohol consumption included ever or current drinkers that drink contain alcohol such as such as beer, wine, spirits, etc.

The differences in the prevalence based on weighted analysis
